# Diffuse skeletal pain after administration of alendronate

**DOI:** 10.4103/0253-7613.68435

**Published:** 2010-08

**Authors:** Nihal Ozaras, Aylin Rezvani

**Affiliations:** Physical Medicine and Rehabilitation Department, Vakif Gureba Training Hospital, Aksaray, Istanbul, Turkey

**Keywords:** Alendronate, biphosphonates, bone pain, musculoskeletal, osteoporosis

## Abstract

**Background:**

Osteoporosis is caused by bone resorption in excess of bone formation, and bisphosphonates, are used to inhibit bone resorption. Alendronate, a biphosphonate, is effective for both the treatment and prevention of osteoporosis in postmenopausal women. Side effects are relatively few and prominently gastrointestinal. Musculoskeletal pain may be an important side effect in these patients. We presented a patient admitted to our out-patient clinic with diffuse skeletal pain after three consecutive administration of alendronate.

**Conclusion:**

We conclude that patients with osteoporosis can report pain, and bisphosphonate-related pain should also be considered before ascribing this complaint to osteoporosis.

## Introduction

Osteoporosis is caused by the cumulative effect of bone resorption in excess of bone formation. Bisphosphonates, one of the available therapeutic options for the management of osteoporosis, inhibit bone resorption. These drugs are also used in the treatment of hypercalcemia, osteoporosis, and Paget’s disease. Alendronate, a biphosphonate, is effective for the treatment and prevention of osteoporosis in postmenopausal women.[1,2] Side effects are relatively few. Transient and mild hypocalcemia and hypophosphatemia are observed in nearly 18 and 10% of the patients, respectively.[1] Gastrointestinal side effect may be prominent especially in patients treated for Paget’s disease at 40 mg per day and include abdominal pain, acid reflux, dyspepsia, nausea, flatulence, diarrhea, gastroesophageal reflux disease, constipation, and abdominal distension. Musculoskeletal pain may be an important side effect in these patients. We present a patient admitted to our out-patient clinic with diffuse skeletal pain after three consecutive administration of alendronate.

## Case Report

A 64-year-old, otherwise healthy woman was evaluated for osteoporosis at our out-patient clinic. On measurement by Dual energy x-ray absorptiometry (DXA) (Lunar), her bone mineral density (BMD) and T-score of L1-L4 lumbar region were found to be 0.789 g/cm^2^ and –3.3, respectively. Alendronate 70 mg weekly and calcium plus vitamin D tablets [1000 mg calcium, 400IU Vitamin D] were prescribed. After taking the medication, she developed a diffuse bone pain throughout the body on the next morning. She used paracetamol 500 mg only once and this pain lasted for 3 days. One week later she took the drug and the pain emerged again that lasted for another 3 days. Although she suspected a relationship between pain and medication, she insisted on taking it. One week later, she took the third dose of alendronate and on next morning she experienced diffuse bone pain again and with a swelling on her right ankle. Then she decided to apply to medical care. On admission erythrocyte sedimentation rate and C-reactive protein levels were elevated. Serum alkaline phosphotase and parathyroid levels were higher than normal levels, while serum calcium level was lower than normal levels. Her alendronate therapy was discontinued and a nonsteroidal anti-inflammatory drug was prescribed. Two weeks later her symptoms resolved and all laboratory all above-mentioned parameters were within normal limits.

## Discussion

Nitrogen-containing bisphosphonates (nBPs) are bone-specific agents that inhibit farnesyl diphosphate synthase. They have a strong affinity for bone, and not for other tissues. Five nBPs are currently approved in the United States. The primary indications are for treatment of osteoporosis (alendronate, ibandronate, and risedronate) and treatment or prevention of skeletal-related events in multiple myeloma and breast and prostate cancer patients (ibandronate, pamidronate, and zoledronic acid). The nBPs reduce osteoporotic fracture risk by 50 to 60% in persons with low bone mass or prior osteoporotic fracture.[3]

Side effects of bisphosphonates are relatively few and usually do not limit the use of them. Bone, joint, and/or muscle pain can be seen during the administration of these drugs and sometimes pain can be attributed to osteoporosis. The Food and Drug Administration reported severe bone, joint, and/or muscle pain, that developed in 112 women, 4 men, 1 adult of unknown sex, and 1 child after starting therapy with alendronate in 2005.[4] The age range as 7 to 84 years (N=109; median=67 years). Bones, joints, and muscles throughout the body were affected. The pain was decribed as “severe,” “extreme,” “disabling,” or “incapacitating.” Many patients were unable to walk, climb stairs, or perform usual activities. Some were reported to be bedridden, and others required walkers, crutches, or wheelchairs. The alendronate doses were 5 mg per day (N=4; 4%); 10 mg per day (N=71; 74%); 20 to 35 mg per day (N=4; 4%); and 70 mg per week (N=17; 18%). The median time to onset of pain was 14 days and pain was treated with a variety of analgesics. Of 83 patients with information, 55 (66%) experienced relief after the drug was discontinued and 9 (11%) of the 83 patients redeveloped pain following readministration of it. In the present case, there was a definite association between the onset of pain and drug administration. The pain reemerged each time the patient took this drug; thereby confirming alendronate as the causal drug for this adverse event.

The patients with osteoporosis can report pain and this complaint can be attributed to the underlying disorders. Not frequently, serious or severe bone, joint, and/or muscle pain begins shortly after bisphosphonate therapy. As in our case, especially re-emergence of the pain after re-challenge should raise the suspicion of an alendronate-associated pain.
